# The Origin and Evolution of the 18th Century Hospital Movement
*The previous article appeared on December 13.


**Published:** 1914-01-17

**Authors:** 


### II. THE ORIGIN AND EVOLUTION OF THE 18th CENTURY HOSPITAL MOVEMENT.*

In 1687 the College of Physicians published an edict enjoining upon all its fellows, candidates, and licentiates to give gratuitous advice, when required, to the neighbouring poor within the City and seven miles, over which their monopoly extended; suggesting as an incentive that by such service they would at one and the same time recommend themselves to the public and improve their knowledge and reputation for success. And in furtherance of the scheme the College somewhat later fitted up a dispensary for the preparation and sale of medicine at cost price to the sick poor, a certain number of the fellows subscribing a sum of 500 guineas for the purposes of the charity.

At its inception the only requirement for admission to the benefits offered by the College was a testimonial from the officiating minister of the parish in which each poor applicant resided; but later, when the support of the Lord Mayor and Aldermen was secured to combat the opposition of the apothecaries, who, finding their monopoly threatened, raised the prices of physic, it was stipulated that the benefits should be extended to those who produced a testimonial from a churchwarden or overseer, and that all hired servants and apprentices to handicraftsmen should for this purpose come within the definition of the poor. (For other purposes the term included only those who came under the care of a parish or workhouse.)

Thus originated the dispensary made famous by the poet-physician Garth, one of its promoters, which performed its functions until 1724, by which time the continuity of the principle upon which it was founded—that of offering to poor applicants the gratuitous advice and prescription of a physician, leaving the patient to procure the medicine at his own cost—had been undertaken by the governors of the Westminster Infirmary. Indeed, it is more than possible that their action prompted the discontinuance of the dispensary. It does not appear to have been recognised in this reference that in 1682, five years previous to the initiation of the London scheme, the College of Physicians of Edinburgh had adopted measures for the medical treatment of the sick poor of

* The previous article appeared on December 13.

that city and its suburbs, thus preparing the way for the principle upon which the dispensary was founded.

In the evolution of the hospital movement of the eighteenth century religious feeling was no unimportant factor. The death of Queen Anne had effected at one stroke the funeral of the Schism Act and the resurrection of religious liberty. Latitudinarianism once again became the order of the day, and the principles of a truer religion inculcating charity and toleration were fostered by the Low Church party and the Nonconformists.

The seventh wave of benevolence was in sight.

In language characteristic of the gentle Friends, John Bellers, a Quaker, advocated in 1714 the erection of hospitals and the provision of adequate medical treatment for the sick poor. And if one can form an opinion from the religious tone of the proposals of the Westminster Charitable Society published in the following year, its promoters —among whom was Mr. Henry Hoare, the banker—belonged to the Low Church party.

The spirit of the times had changed since the days when the Royal hospitals were founded in order to protect the public from the dangers of contact "with the miserable people lying in every street and offending every clean person passing by the way with their filthy and nasty savours." The motive of securing the protection of the healthy had given way to the desire to alleviate the sufferings of the sick; measures which had been introduced from the standpoint of public expediency were continued and re-established upon humanitarian principles. Compare with the above extract from the petition of Sir Richard Gresham to Henry VIII. the charitable proposal of the Westminster Society which may be regarded as the firstfruits of Bellers' suggestions.

"Notwithstanding the provision settled by our laws and the collections made by the charity of well-disposed Christians for the relief of the poor, it is obvious to anyone that walks the streets that the same is not sufficient to preserve great numbers of them from begging to the great grief of all good men, and the no small reproach of our religion and country, etc. Frequent attempts have been made to provide for the poor, but hitherto they have been ineffectual; they must depend upon the voluntary assistance of charitable people and a Christian spirit on the part of those persons employed to take care of the poor, etc." The intention was to provide the sick with food and physic during illness; to procure for them the advice of physicians and the assistance of surgeons and nurses; to supply lying-in women with the necessary lodging and attendance; to visit and relieve sick prisoners; to furnish money for the return to their own country of destitute strangers; and to obtain the ministrations of the clergy for the reclamation of the souls of the outcast.

It was intended to establish branches in distant parts of the city, and, if successful, in every parish; to engage repositories for the storage of supplies of food, clothing, linen, bedding, furniture, etc.; and to obtain the services of leisured persons to collect subscriptions and to visit, tend, and comfort the sick and destitute.

The Society started upon its charitable project in January 1715-6, but before three months had passed its financial position was so unsatisfactory that it became necessary to limit its benefits to the residents of St. Margaret's parish, shortly after which it came to an untimely end for want of funds. Brief though its existence, it is of importance, as being the earliest charitable society of which we have detailed cognisance, to be established for the provision of medical treatment for the sick poor, and supported by voluntary subscriptions; and it forms a connecting link in the chain of sick relief dispensed by the early guilds, by the religious houses, by the Church collections for the impotent poor ordered by the Statute of 1536, by the Royal foundations of St. Bartholomew and St. Thomas, and by the general hospitals of modern times.

In his history of the London Hospital Mr. Morris asks the questions:—

Why, after 500 years of apparent neglect, there should have been this sudden awakening (in the eighteenth century) to the needs of the sick poor?

Was there some sudden advance in medicine and surgery in the benefits of which the rich considered that the poor should have their share?

Was there some great wave of public philanthropy?

Was it a time of great sickness and of great poverty?

Or were the hospitals founded for reasons which must be described as selfish? Were the sick poor herded together because it was better for the sick poor, or because it was much better for the non-sick rich?

But that the hospital movement was no sudden development, no impulse of the moment, becomes apparent when its history is fully considered. The dominant principle of providing for the gratuitous treatment of the sick poor, first seen in the Edinburgh and dispensary schemes, in which it takes the form of the advice of a physician, with medicines at cost price, makes its reappearance in the proposals of John Bellers advocating the erection of hospitals (the major part of the expense being borne by the State, the minor by voluntary contributions), and the engagement of a physician, surgeon, and apothecary in every parish (at the cost of the rates), is found again in practical form in the measures carried out by the Westminster Charitable Society, and at length reached its consummation in the establishment of the first voluntary hospital for the reception of the sick *free of all expense.*

The immediate and exciting cause of the latter development lies hidden in the italics. Outside the few workhouse infirmaries, no public accommodation was then available for the sick poor except the two chartered hospitals, which had become quite inadequate at a peculiarly distressful period to the needs of the then population. The imposition at those institutions of a demand for fees from all applicants for admission acted as a class limitation by excluding those in the direst need, who were unable to procure the necessary money, yet were ineligible from one cause or another for parish relief. For them no alternative was left but unrelieved suffering, yet they were the very persons for whom the chartered hospitals were originally intended.

A detailed study of all the circumstances goes far to show that it was the imposition of these fees which suggested to certain charitable persons the necessity of immediate action; and since the unsettled state of the country amid the uncertainty and dangers of a disputed succession negatived any hope of the State-aid postulated in the proposals of John Bellers, the small band of Westminster philanthropists headed by Henry Hoare appealed once again for the voluntary subscriptions and benefactions of the public, this time for the purpose of establishing and maintaining an infirmary for the *reception* and treatment of the destitute sick, free of all expense.

At last success attended his efforts and, late though it be, his name, already the subject of varied encomium, should henceforth be set aloft as that of the man above all others to whose benevolence, zeal, and perseverance our voluntary hospital system owes its origin.

